# Lecture on Physical Exercise

**Published:** 1885-03

**Authors:** 


### A LECTURE ON PHYSICAL EXERCISE.

"I am continually asked,' said Dr. Sargent, "'What is the best exercise for girls?' or 'What is the best popular exercise?' Nearly every one had a popular exercise, but no single exercise developed every muscle of the body," he went on to say. "The first exercise acquired, that of walking, was first spoken of. Arduous as it was to acquire, when once acquired it becomes one of the simplest of exercises. The muscles employed in walking were then pointed out by the lecturer. He had traced a number of cases of heart disease in boys who took long walks with their parents. This was caused by the boys being obliged to run to keep up with the older walker. Running as an exercise was next taken up. Strong, rapid runners always had large development of the thigh muscles. This exercise should be taken with great care by men 30 years of age or so. It was better to take a quick, short run than an extended one. Swimming brought more muscles into action than any other exercise. There was resistance everywhere. It was a great pity that Boston was not provided with large swimming baths. It was a great prevention of lung troubles, and too much stress could not be laid upon its value as an exercise. The next important of general exercise was that of rowing. The abdominal muscles assisted in drawing the body forward in rowing, but the thigh muscles also contributed to this action. The lecturer then picked out several errors practiced by oarsmen in regard to the economic employment of muscular force. The disadvantages of rowing with a straight back were emphasized. The theory that rowing did nothing for the chest was slightly in error. As a usual thing, the expansive extent of the respiratory organs was fully as large as in most athletes.

"Skating, as practiced in the roller skating rinks, is an admirable exercise for gaining poise and grace. The lecturer gave warning against overdoing it, however. The invention of the bicycle was a boon to the American people. Its special beauties were the opportunities which it gave for change of air and scene. It was more of an exercise than walking for the legs. There were tendencies in bicycle riders, such as bending over, which were dangerous. But this was not necessary. It was one of the best exercises for developing the extensor muscles of the thigh. It gave strength to the knee joint also.

"The reason why the bicycle made some young men round-shouldered was because the handle-bars were too near, or the rider was too much inclined to speeding. The tricycle was better for older men. Football, he was free to confess, had no peer as a general developer, and for that reason he was sorry that it has been allowed to degenerate into a brutal contest. It could be played in a gentlemanly way, he thought, and still do great good. Wonderful development arose from the practice of the game. Sparring was a particularly good exercise for the respiratory organs, eyes and muscles of the body. It was something every American should know. It was something honorable for two men to stand up and spar in a fair manner. The exercise had a tendency to stir up sluggish systems, and kept a person's mind in equal play with the muscles. Sparring could be made brutal, and he was sorry to say that that was the tendency of all antagonistic sports When sparring clubs met to witness glove contests, then degeneracy began. Wrestling, as an exercise for the muscles of the back, chest and waist, was admirable. It was always attended with danger. Archery had a good deal to commend its practice, especially for young girls. The exercise naturally made a person straight in figure. The almost invariable straightness of Indians was caused by the use of the bow. Military drill as an exercise was a misnomer. There is no freedom of movement, and all military movements were cramped. His shoulders, projecting hip, etc., marked the victim of military dress. In France and Germany exercises are in vogue in order to correct the bad effects of the drill. Marching, as a part of the drill, was excellent, however. Other forms of gymnastic exercise, such as Indian clubs, pulley weights, etc., were spoken of, and their good and bad points as exercises pointed out."—*Boston Herald.*

